# Systematic identification of aberrant non-coding RNAs and their mediated modules in rotator cuff tears

**DOI:** 10.3389/fmolb.2022.940290

**Published:** 2022-08-30

**Authors:** Yichong Zhang, Jianhai Chen, Shengyuan He, Yun Xiao, Aiyu Liu, Dianying Zhang, Xia Li

**Affiliations:** ^1^ Department of Orthopedics and Trauma, Key Laboratory of Trauma and Neural Regeneration (Ministry of Education/Peking University), Peking University People’s Hospital, Beijing, China; ^2^ College of Bioinformatics Science and Technology, Harbin Medical University, Harbin, Heilongjiang, China; ^3^ Central Laboratory, Peking University People’s Hospital, Beijing, China

**Keywords:** rotator cuff tears, miRNA, lncRNA, circRNA, ceRNA network

## Abstract

**Background:** Rotator cuff tears (RCT) is the most common cause of shoulder dysfunction, however, its molecular mechanisms remain unclear. Non-coding RNAs(ncRNAs), such as long ncRNA (lncRNA), microRNA (miRNA) and circular RNA (circRNA), are involved in a variety of diseases, but little is known about their roles in RCT. Therefore, the purpose of this study is to identify dysregulated ncRNAs and understand how they influence RCT.

**Methods:** We performed RNA sequencing and miRNA sequencing on five pairs of torn supraspinatus muscles and matched unharmed subscapularis muscles to identify RNAs dysregulated in RCT patients. To better comprehend the fundamental biological processes, we carried out enrichment analysis of these dysregulated mRNAs or the co-expressed genes of dysregulated ncRNAs. According to the competing endogenous RNA (ceRNA) theory, we finally established ceRNA networks to explore the relationship among dysregulated RNAs in RCT.

**Results:** A total of 151 mRNAs, 38 miRNAs, 20 lncRNAs and 90 circRNAs were differentially expressed between torn supraspinatus muscles and matched unharmed subscapularis muscles, respectively. We found that these dysregulated mRNAs, the target mRNAs of these dysregulated miRNAs or the co-expressed mRNAs of these dysregulated ncRNAs were enriched in muscle structure development, actin-mediated cell contraction and actin binding. Then we constructed and analyzed the ceRNA network and found that the largest module in the ceRNA network was associated with vasculature development. Based on the topological properties of the largest module, we identified several important ncRNAs including *hsa_circ_0000722*, hsa-miR-129-5p and hsa-miR-30c-5p, whose interacting mRNAs related to muscle diseases, fat and inflammation.

**Conclusion:** This study presented a systematic dissection of the expression profile of mRNAs and ncRNAs in RCT patients and revealed some important ncRNAs which may contribute to the development of RCT. Such results could provide new insights for further research on RCT.

## 1 Introduction

The rotator cuff consists of four muscle-tendon units: supraspinatus, infraspinatus, teres minor and subscapularis, which contribute to shoulder movement. Rotator cuff tear (RCT) is the leading cause of pain and functional disability of the shoulder and is present in about 30% of individuals in their 60 s and higher in individuals over 80 years old (Dang and Davies, 2018). Although some patients can be treated successfully with surgical repair (Schemitsch et al., 2019), not all patients’ outcomes of rotator cuff repair are satisfactory, which is partly because of the poor understanding of the molecular mechanism of RCT (Connor et al., 2019). Therefore, potential factors that may contribute to RCT should be identified.

Non-coding RNAs (ncRNAs) account for approximately 97% of the human genome, including long ncRNAs (lncRNA), microRNAs (miRNA) and circular RNAs (circRNA) (Anastasiadou et al., 2018). LncRNAs are a class of ncRNAs longer than 200 bp with low coding potential, while miRNAs are a class of small ncRNAs with ∼22 nucleotides. They were reported to influence various stages of tendinopathy and could be implicated in skeletal muscle differentiation (Ge et al., 2020a; Plachel et al., 2020). CircRNAs are a novel class of endogenous, non-coding RNAs with closed-loop structures, which were generated during RNA alternative splicing. CircRNAs can express in striated muscle tissues including skeletal and cardiac muscles as well (Greco et al., 2018). LncRNAs and circRNAs can act as molecular sponges of miRNAs to regulate the expression of mRNAs, which is known as “competing endogenous RNA (ceRNA)” hypothesis. Previous studies have shown that lncRNAs and circRNAs could serve as ceRNAs and play roles in rotator cuff tendinopathy (Ge et al., 2020a; Ge et al., 2020b). However, the research of lncRNA- or the circRNA-mediated ceRNA networks is not comprehensive.

In this study, we screened and identified differentially expressed mRNAs, lncRNAs, circRNAs and miRNAs between samples from torn supraspinatus and unharmed subscapularis. Based on the results of differential expression analysis and miRNA targeting information, we constructed lncRNA/circRNA-associated dysregulated ceRNA networks in RCT. Finally, we found the largest module in the ceRNA network and identified several important ncRNAs in this module, which may have roles in RCT.

## 2 Results

### 2.1 Identification of differentially expressed mRNAs

**FIGURE 1:**
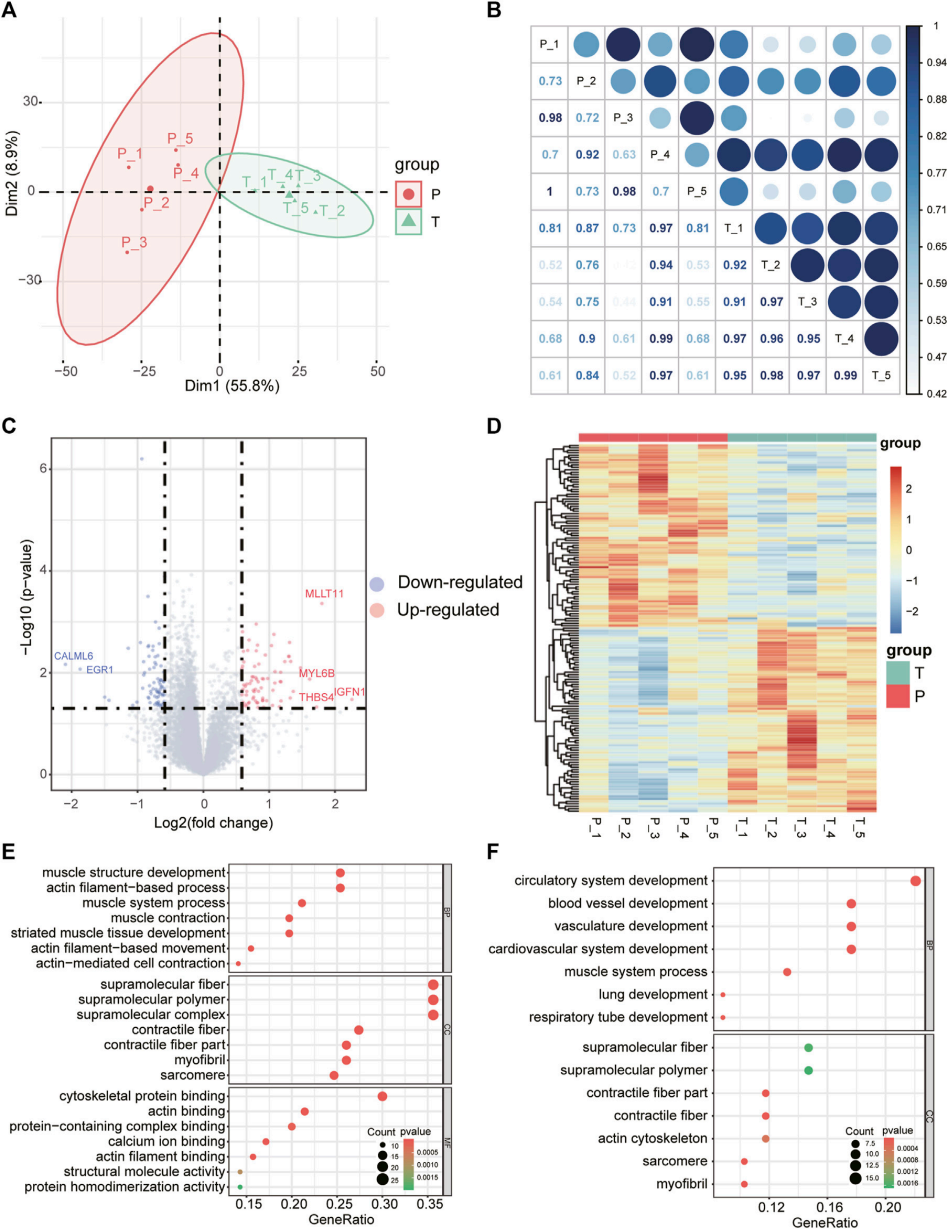
Differentially expressed mRNAs and GO enrichment analysis. **(A)** The first two principal components show distinction between torn supraspinatus muscles and matched unharmed subscapularis muscles based on the mRNA expression profile. **(B)** Heatmap of the correlation matrix of mRNA expression profile of 10 samples, the size of the node represents the correlation coefficient. **(C)** Volcano plot of differentially expressed mRNAs in RCT. Red points denote up-regulated mRNAs, and blue points denote down-regulated mRNAs. The most up- or down-regulated mRNAs are labeled. **(D)** Heatmap depicting expression levels of the differentially expressed mRNAs in RCT. **(E,F)** GO enrichment analysis for the up-regulated**(E)** and down-regulated differentially expressed mRNAs **(F)**, respectively.

RNA sequencing was performed to obtain the mRNA expression profile of torn supraspinatus muscles (group T) and matched unharmed subscapularis muscles (group P). Principal component analysis of the mRNA expression data could distinguish samples of supraspinatus muscles and unharmed subscapularis muscles, indicating that the difference between supraspinatus muscles and unharmed subscapularis muscles (Figures 1A,B). Compared with the expression of mRNAs in unharmed subscapularis muscles, a total of 151 differentially expressed mRNAs in supraspinatus muscles (absolute fold change >1.5 and *p* < 0.05) were identified, which comprised 76 up-regulated mRNAs and 75 down-regulated mRNAs (Figures 1C,D). Some of these differentially expressed mRNAs were mentioned in previous reports of rotator cuff tears. For example, *MYL6B* has been proved that it can differentially expressed in patients with rotator cuff tears (Frich et al., 2021), while *EGR1* is conducive to the repair of rotator cuff tears (Tao et al., 2015). We further applied enrichment analysis on the differentially expressed mRNAs. The results showed that differentially expressed mRNAs enriched in muscle structure development, actin filament-based process and blood vessel development (Figures 1E,F). Previous researches also indicated that rotator cuff tears could be associated with muscle actin and angiogenesis (Fuchs et al., 2008; Noh et al., 2018). After adjusting the P value using the false discovery rate (FDR) method, only one differentially expressed mRNA, *SIM2*, was identified (FDR corrected *p* < 0.05).

### 2.2 Identification of differentially expressed ncRNAs

Studies have characterized the biological roles of noncoding RNAs in many diseases (Falcone et al., 2014; Li et al., 2017; Kristensen et al., 2019; Li et al., 2021), therefore, we also performed miRNA sequencing on torn supraspinatus muscles and matched unharmed subscapularis muscles. Together with the previous data of RNA sequencing, we generated the expression profiles of miRNAs, lncRNAs and circRNAs.

**FIGURE 2:**
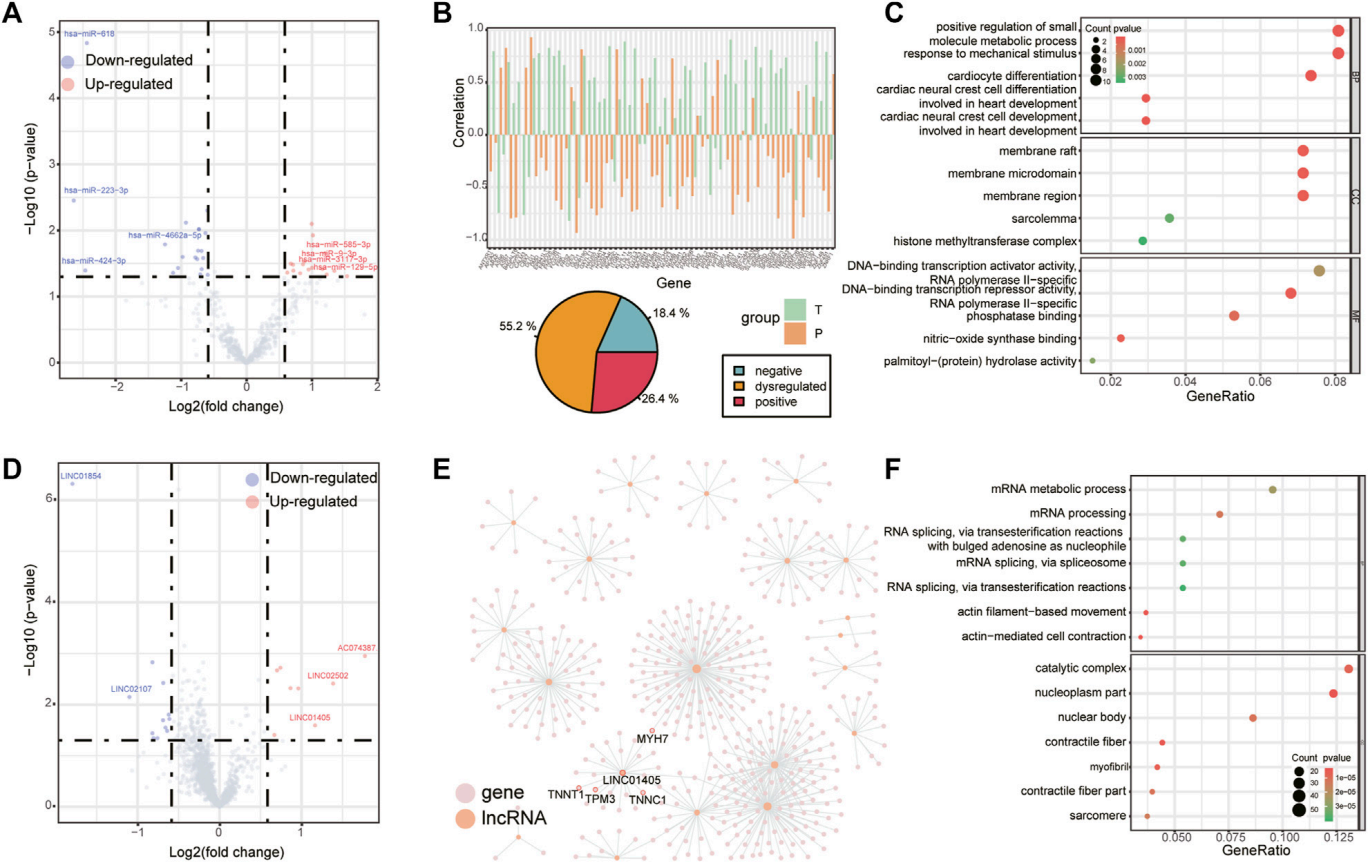
Differentially expressed miRNAs and lncRNAs. **(A)** Volcano plot of differentially expressed miRNAs in RCT. Red points denote up-regulated miRNAs, and blue points denote down-regulated miRNAs. The most up- or down-regulated miRNAs are labeled. **(B)** Changes in the correlation between hsa-miR-9-3p and its target genes, bar chart (top panel) shows the correlation of those miRNA-mRNA pairs which were positively (negatively) correlated to hsa-miR-9-3p in supraspinatus muscles but negatively (positively) correlated to hsa-miR-9-3p in unharmed subscapularis muscles (we define this correlation pattern as “dysregulated”), pie chart (bottom panel) shows the proportion of miRNA-mRNA pairs with both negative correlation, both positive correlation or “dysregulated” in supraspinatus muscles and unharmed subscapularis muscles. **(C)** GO enrichment analysis for all target mRNAs of hsa-miR-9-3p. **(D)** Volcano plot of differentially expressed lncRNAs. **(E)** Co-expression network of differentially expressed lncRNAs. Pink points denote mRNAs and orange points denote lncRNAs. **(F)** GO enrichment analysis for all co-expressed mRNAs of differentially expressed lncRNAs.

**FIGURE 3:**
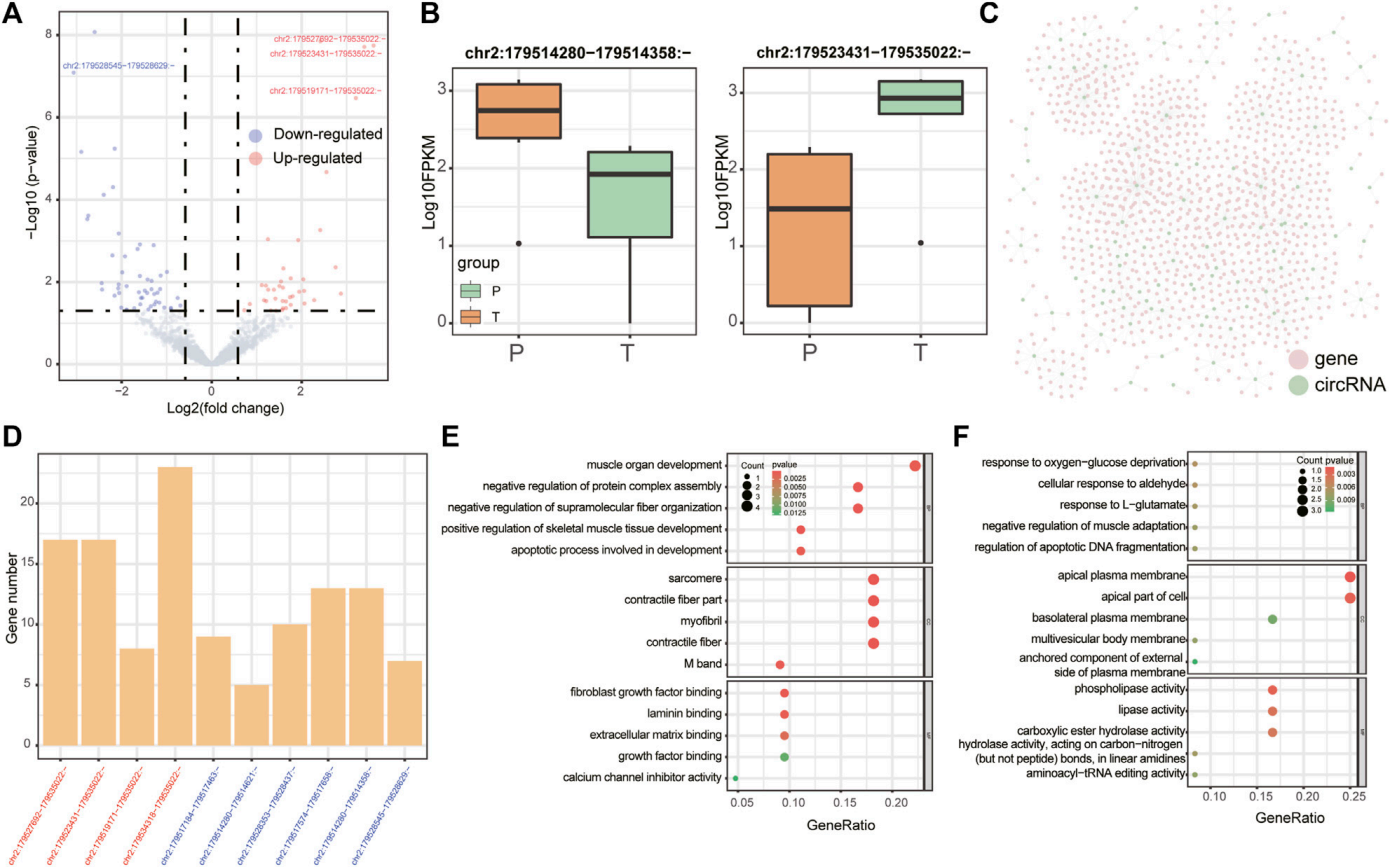
Differentially expressed circRNAs. **(A)** Volcano plot of differentially expressed circRNAs in RCT. Red points denote up-regulated circRNAs, and blue points denote down-regulated circRNAs. The most up- or down-regulated circRNAs are labeled. **(B)** Boxplots denote expression levels of 2 differentially expressed circRNAs which were derived from *TTN* (*chr2:179514280–179514358:-* (left panel) and *chr2:179523431–179535022:-* (right panel)). **(C)** Co-expression network of differentially expressed circRNAs. Pink points denote mRNAs and green points denote circRNAs. **(D)** Barplots denote the numbers of mRNAs regulated by circRNAs that were produced by *TTN* in the co-expression network. CircRNAs were ordered according to the fold changes in group T vs. group P (all circRNAs were identified both by edgeR and by GLMM). **(E,F)** GO enrichment analysis for all co-expressed mRNAs of *chr2:179534318–179535022:-* and *chr2:179514280–179514358:-*.

There were 38 differentially expressed miRNAs (17 up-regulated and 21 down-regulated, Figure 2A), 20 differentially expressed lncRNAs (8 up-regulated and 12 down-regulated, Figure 2D) and 90 differentially expressed circRNAs (39 up-regulated and 51 down-regulated, Figure 3A) were identified in supraspinatus muscles and unharmed subscapularis muscles. After adjusting the P value using the FDR method, only 1 differentially expressed miRNA (hsa-miR-618), 1 differentially expressed lncRNA (*LINC01854*) and 15 differentially expressed circRNAs (*chr2:152355811–152355904:-*, *chr2:179511211–179511286:-*, *chr2:179514280–179514358:-*, *chr2:179514280–179514621:-*, *chr2:179517184–179517463:-*, *chr2:179517574–179517658:-*, *chr2:179519171–179535022:-*, *chr2:179523431–179535022:-*, *chr2:179527692–179535022:-*, *chr2:179528353–179528437:-*, *chr2:179528545–179528629:-*, *chr2:179534318–179535022:-*, *hsa_circ_0141770*, *chr2:179542347–179544143:-*, *hsa_circ_0086735*) were identified (FDR corrected *p* < 0.05).

One of the differentially expressed miRNA, hsa-miR-9-3p (Figure 2A), may be involved in inflammation (Chakraborty et al., 2015), which is a common symptom in rotator cuff tears. We compared the correlation between the expression of hsa-miR-9-3p and its target mRNAs in supraspinatus muscles and unharmed subscapularis muscles. More than half of the target mRNAs of hsa-miR-9-3p were positively (negatively) correlated to hsa-miR-9-3p in supraspinatus muscles but negatively (positively) correlated to hsa-miR-9-3p in unharmed subscapularis muscles (Figure 2B). Such opposite correlation results in supraspinatus muscles and unharmed subscapularis muscles support the opinion that hsa-miR-9-3p was dysregulated in rotator cuff tears. We also found that target mRNAs of hsa-miR-9-3p were enriched in positive regulation of small molecule metabolic process, response to mechanical stimulus, membrane raft and sarcolemma (Figure 2C).

Next, we performed co-expression analysis to identify mRNAs correlated to differentially expressed lncRNAs (Figure 2E). Previous study had reported that one of the differentially expressed lncRNAs, *LINC01405*, was associated with muscle-related disease (Schofer et al., 2008). Some co-expressed mRNAs of *LINC01405* (*TNNT1* and *MYH7*, Figure 2E) have also been reported in a study of rotator cuff tears (Frich et al., 2021). Overall, co-expressed mRNAs of all differentially expressed lncRNAs were enriched in actin-mediated cell contraction and contractile fiber (Figure 2F).

Meanwhile, we also analyzed differentially expressed circRNAs in supraspinatus muscles and unharmed subscapularis muscles. In addition to applying edgeR, we employed the GLMM model, a newly developed approach specifically for identifying differentially expressed circRNAs (Buratin et al., 2022). A total of 28 differentially expressed circRNAs were identified by GLMM model (*p* < 0.05 FDR corrected), and 22 of them overlapped with the results identified by edgeR (*p* = 0.007, hypergeometric test). After adjusting the p-value with FDR <0.05, there was significant overlap of 10 circRNAs between the results of edgeR and GLMM (p = $2.92\times 10^{-16}$ , hypergeometric test).

All 10 differentially expressed circRNAs identified both by edgeR and by GLMM were derived from *TTN*(Titin) (Figures 3B,C). An earlier study has found that the molecular weight of *TTN* was changed in the injured rotator cuff (Sato et al., 2014). Although these *TTN*-derived circRNAs were derived from the same gene, we found that different functions were related to them, which also co-expressed with a various number of mRNAs (Figure 3D). For example, co-expressed mRNAs of *chr2:179534318–179535022:-* were enriched in muscle organ development and skeletal muscle tissue development (Figure 3E), while co-expressed mRNAs of *chr2:179514280–179514358:-* were associated with negative regulation of muscle adaptation, response to oxygen-glucose deprivation (Figure 3F).

### 2.3 Establishing dysregulated ceRNA networks of lncRNAs and circRNAs

**FIGURE 4:**
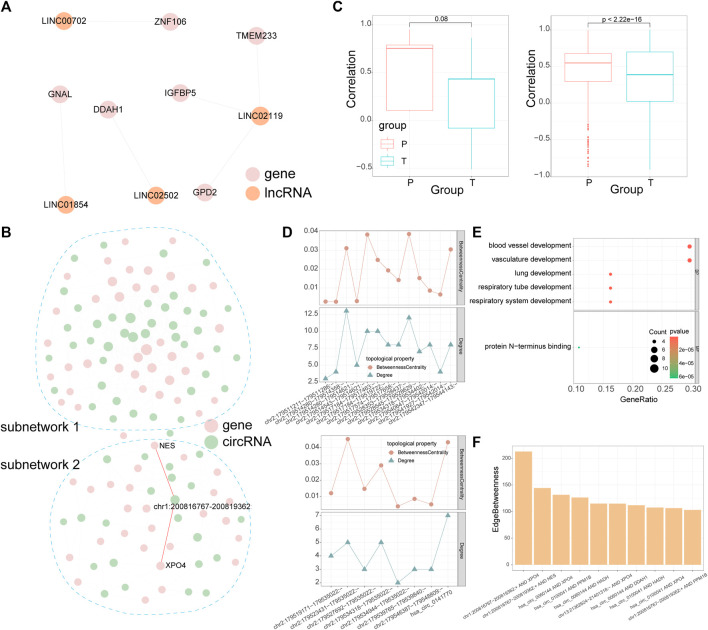
CeRNA networks of lncRNA and circRNA. **(A)** The dysregulated ceRNA network of lncRNAs. The size of the node represents the node’s degree. **(B)** The dysregulated ceRNA network of circRNAs. CircRNA-mRNA pairs with the highest edge betweenness are highlighted in red. **(C)** Pearson correlation coefficient between lncRNA/circRNA-mRNA pairs in the dysregulated ceRNA networks of lncRNAs (left panel) or circRNAs (right panel). **(D)** Line chart denotes the degree and betweenness centrality of mRNAs regulated by circRNAs which were produced by *TTN* in subnetwork 1 (top panel) and subnetwork 2 (bottom panel). **(E)** GO enrichment analysis for mRNAs in subnetwork 1. **(F)** Edge betweenness centrality of circRNA-mRNA pairs in subnetwork 2, only the top 10 are shown in the diagram. In the dysregulated ceRNA network, pink points denote mRNAs, orange points denote lncRNAs and green points denote circRNAs.

LncRNAs can act as molecular sponges of miRNAs to regulate the expression of mRNAs and this mechanism is known as “competing endogenous RNA (ceRNA)” hypothesis. According to the ceRNA hypothesis, a large number of studies have explored the lncRNAs-miRNAs-mRNAs interactions in various diseases which include rotator cuff tears (Ge et al., 2020a; Plachel et al., 2020; Chen et al., 2021). Additional to lncRNAs, recent studies also showed that circRNAs could serve as ceRNAs, however, there has been few studies characterize circRNAs-miRNAs-mRNAs interactions in rotator cuff tears. We obtained target mRNAs and lncRNAs/circRNAs from the starbase, mirTarbase, and lncbase; then we identified lncRNAs/circRNAs-mRNAs pairs which significantly share common miRNAs by the hypergeometric test based on ceRNA hypothesis (see Section 5, Figures 4A,B). As a result, the dysregulated lncRNA-mRNA ceRNA network included five lncRNAs and 8 mRNAs, while the dysregulated circRNA-mRNA ceRNA network included 65 circRNAs and 65 mRNAs. The distribution of correlation coefficients between the expression of lncRNAs/circRNAs and mRNAs in the dysregulated network was different in torn supraspinatus muscles and matched unharmed subscapularis muscles (Figure 4C), which also indicate the dysregulation of ceRNA interaction in rotator cuff tears.

In the dysregulated ceRNA network of circRNAs, we detected two subnetworks (Figure 4B). These two subnetworks had a various number of *TTN*-derived circRNAs, whose degree and betweenness centrality varies in each subnetwork as well (Figure 4D). We further performed enrichment analysis on mRNAs in each subnetwork and calculated the edge betweenness centrality of each circRNA-mRNA pair in these two subnetworks. MRNAs in the subnetwork 1 were related to vasculature development and lung development by enrichment analysis (Figure 4E). Although there were no GO terms enriched in mRNAs in the subnetwork 2, the 2 circRNA-mRNA pairs with the highest edge betweenness centrality in subnetwork 2 were both mediated by a *CAMSAP2*-derived circRNA (*chr1:200816767–200819362:+*, Figures 4B,F). Some studies indicated that *CAMSAP2* was associated with microtubule (Li et al., 2020), which could take part in muscle differentiation (Gache et al., 2017).

### 2.4 Identifying the key ncRNA mediating the ceRNA module

**FIGURE 5:**
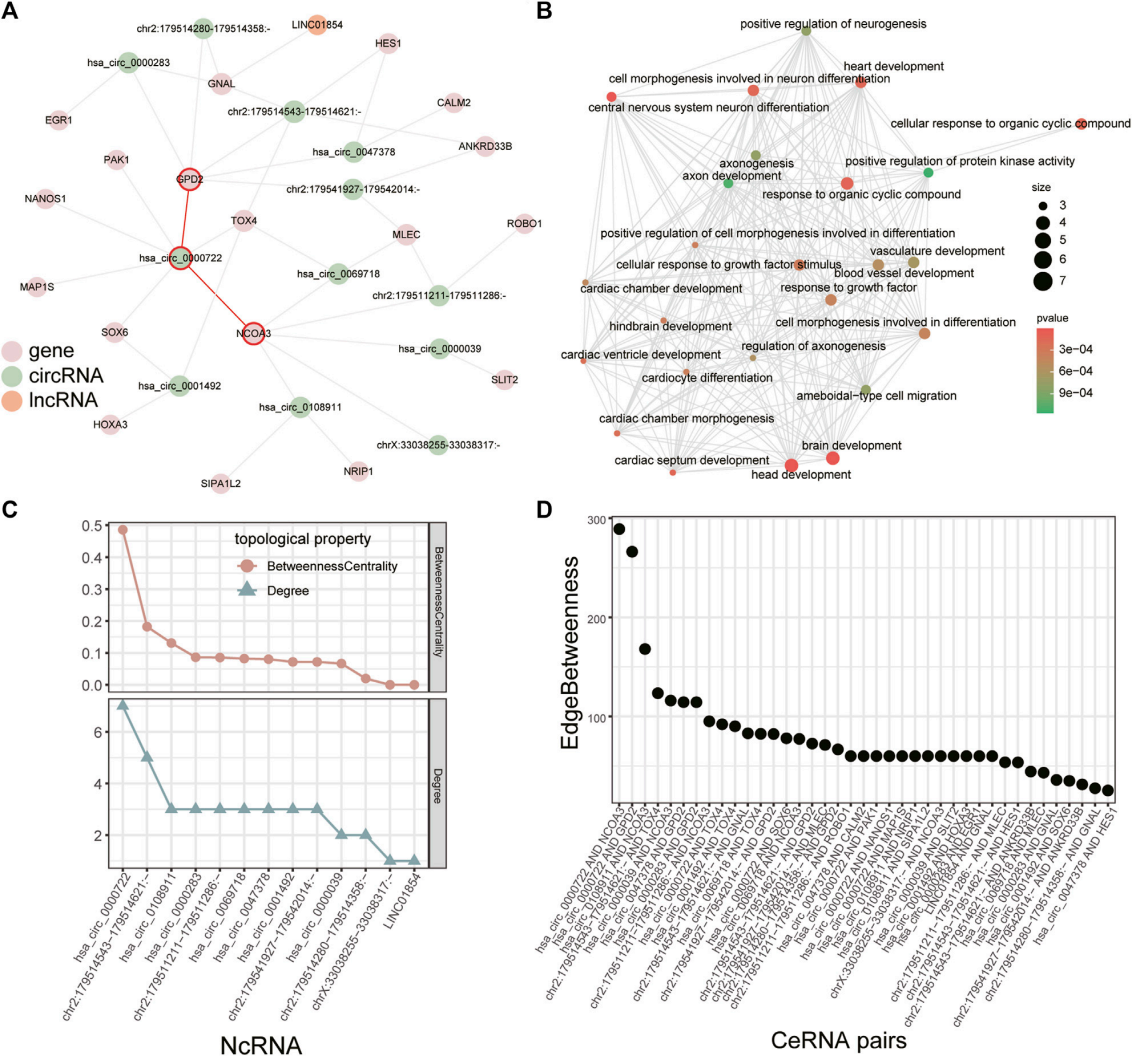
Module in the combined ceRNA network. **(A)** The largest module in the combined ceRNA network. The circRNA-mRNA pairs of *hsa_circ_0000722* are highlighted in red. **(B)** GO enrichment analysis for the mRNAs in the module. **(C)** Distribution of degree and betweenness centrality of ncRNAs in the module. **(D)** Edge betweenness centrality of lncRNA/circRNA-mRNA pairs in the module.

Module analysis of the dysregulated ceRNA network can provide more information about rotator cuff tears. As lncRNAs and circRNAs both can influence mRNAs by competing for shared miRNAs, we combined the dysregulated lncRNA-mRNA ceRNA network with the dysregulated circRNA-mRNA ceRNA network according to mRNAs in both networks (see Section 5). The combined network included five lncRNAs, 65 circRNAs and 67 mRNAs, then we used “clusterMaker2” (Morris et al., 2011) to identify modules in the combined network. The largest module had 12 circRNAs, 1 lncRNA and 18 mRNAs (Figure 5A). Further analysis showed that mRNAs in the largest module were associated with vasculature development and heart development (Figure 5B).

We identified key ncRNAs by calculating the degree and betweenness centrality of each node in the largest module. *Hsa_circ_0000722* had the largest degree and betweenness centrality (Figure 5C) and recent study has revealed that *hsa_circ_0000722* has many potential binding sites for genes of MBNL family, a gene family which can cause muscle disease (Czubak et al., 2019). Based on the edge betweenness centrality of each lncRNA/circRNA-mRNA pair in this module (Figure 5D), we found that the lncRNA/circRNA-mRNA pairs with the highest edge betweenness centrality were mediated by *hsa_circ_0000722* as well (*hsa_circ_0000722*-*GPD2* and *hsa_circ_0000722*-*NCOA3*, Figures 5A,D)*.* Studies have shown that *GPD2* and *NCOA3* relate to the fat (Mollah and Ishikawa, 2010; Han et al., 2017), which could infiltrate in injured rotator cuff muscles (Khanna et al., 2019).

### 2.5 Identifying the key miRNAs regulating the ceRNA module

**FIGURE 6:**
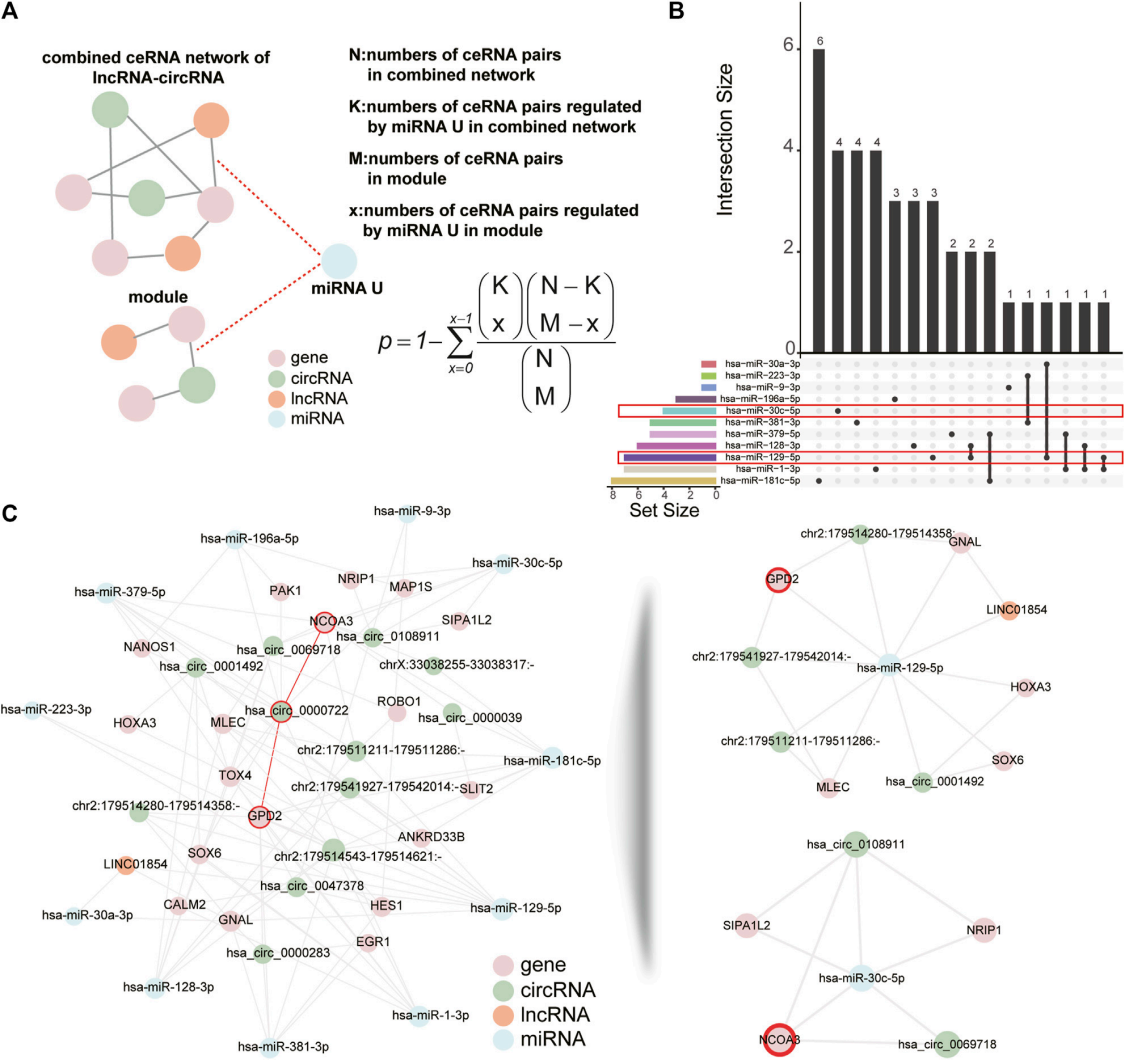
Critical miRNAs associated with the module. **(A)** Schematic of identifying critical miRNAs that significantly regulate lncRNA/circRNA-mRNA pairs in the module. Dashed lines denote miRNA targeting relationship and brown solid lines denote ceRNA relationship. **(B)** The UpSet plot demonstrates overlap in lncRNA/circRNA-mRNA pairs which are mediated by miRNAs in the ceRNA module. The 2 critical miRNAs are highlighted in red. **(C)** Visualization of the module with miRNAs (left panel) and lncRNA/circRNA-mRNA pairs regulated by the two critical miRNAs (right panel). The circRNA-mRNA pairs and mRNAs related to *hsa_circ_0000722* are highlighted in red.

Given that each ceRNA shares multiple miRNAs with numerous other ceRNAs, we then attempted to identify the key miRNAs in the largest ceRNA module (Figure 6A). A total of 11 differentially expressed miRNAs were associated with the ceRNA module and the lncRNA/circRNA-mRNA pairs they mediated were quite different (Figure 6B). Based on the hypergeometric test, we could identify which miRNAs mediated ceRNA pairs more significantly in the largest module than in the combined network. Finally we detected 2 important miRNAs: hsa-miR-129-5p and hsa-miR-30c-5p (p-value < 0.05, Figures 6A,C, see Section 5). The *hsa_circ_0000722*-*GPD2* and *hsa_circ_0000722*-*NCOA3* pairs that we identified above were mediated by hsa-miR-129-5p (*hsa_circ_0000722*-hsa-miR-129-5p-*GPD2*) and hsa-miR-30c-5p (*hsa_circ_0000722*-hsa-miR-30c-5p-*NCOA3*), respectively (Figure 6C). Some target mRNAs of hsa-miR-129-5p or hsa-miR-30c-5p, such as *GNAL*, *SOX6* and *NRIP1*, have been demonstrated to be associated with muscle diseases (Connor et al., 1995; Hagiwara et al., 2000; Kumar et al., 2014; De Marinis et al., 2017). *Hsa_circ_0108911* is derived from ATP9B and is also a target circRNA of hsa-miR-30c-5p. Although there were no studies about *hsa_circ_0108911*, Sun et al. (2020) have shown that another ATP9B-derived circRNA, circAtp9b, can contribute to the inflammation.

## 3 Discussion

In this study, we comprehensively dissected the dysregulated transcriptome of RCT, including mRNAs, miRNAs, lncRNAs, and circRNAs. According to the ceRNA hypothesis, we constructed the dysregulated ceRNA network and identified several important ncRNAs in the largest module of the ceRNA network, including *hsa_circ_0000722*, hsa-miR-129-5p and hsa-miR-30c-5p.

We have obtained transcripts that were dysregulated in the RCT by identifying differentially expressed mRNAs/ncRNAs between torn supraspinatus muscles and normal supraspinatus muscles. However, the transcripts that we identified may also show the inherent differences between supraspinatus muscles and subscapularis muscles. Recent studies have investigated the properties of different muscles by identifying differentially expressed genes between different muscles (Alto et al., 2021; Smith et al., 2022); Terry et al. revealed that an average of 13% of transcripts were differentially expressed between any two skeletal muscles (Terry et al., 2018). Therefore, further transcriptome sequencing of normal supraspinatus muscles and normal subscapularis muscles is required to confirm that the dysregulation of these transcripts is associated with RCT rather than being caused by tissue differences.

It is noted that all of differentially expressed circRNAs identified both by edgeR and by GLMM were derived from *TTN*, a gene which encodes proteins of striated muscle and contributes to muscle contraction (Hessel et al., 2017). Such a number of *TTN*-derived circRNAs may be due to the giant size and complexity of *TTN* (Chauveau et al., 2014). Because of the important role of *TTN* itself in muscle, the *TTN*-derived circRNAs are also more likely to be involved in muscle-related functions, which means further studies are needed to demonstrate the effect of these *TTN*-derived circRNAs in rotator cuff tears.

Functional enrichment analysis of the dysregulated transcriptome revealed several functions which may be associated with rotator cuff tears, such as muscle contraction, vasculature development, lung development and heart development. Studies have shown that muscle contraction of the rotator cuff plays an important role in moving and stabilizing the glenohumeral joint (Edouard et al., 2011). Since increased vascularization around the tendon-bone interface is essential for promoting rotator cuff tendon-bone healing (Randelli et al., 2016; Noh et al., 2018), angiogenesis is a key process after repairing of rotator cuff tears. In addition to these studies, some studies also explored the potential association of rotator cuff tears with lung diseases and cardiovascular system (Bachasson et al., 2015; Nam et al., 2015; Applegate et al., 2017). Taken together, these results could serve as a reference for future studies of rotator cuff tears.

We identified an important ncRNA (*hsa_circ_0000722*) by analyzing the dysregulated ceRNA networks of rotator cuff tears. Several mRNAs or ncRNAs which interact with *hsa_circ_0000722* in the largest ceRNA module, including *GPD2*, *NCOA3* and hsa-miR-30c-5p, are associated with fat (Mollah and Ishikawa, 2010; De Marinis et al., 2017; Han et al., 2017; Yaman et al., 2021). Hsa-miR-129-5p and hsa-miR-30c-5p were the other 2 key ncRNA we identified, which all interact with *hsa_circ_0000722* in the ceRNA network (*hsa_circ_0000722*-hsa-miR-129-5p-*GPD2*/*hsa_circ_0000722*-hsa-miR-30c-5p-*NCOA3*). These 2 miRNAs have been found to be dysregulated in inflammation-related diseases, implying that they may involve in the inflammatory response in RCT (Duecker et al., 2022; Ling et al., 2022). Studies showed that inflammation in RCT contributes to fatty infiltration (Nelson et al., 2021), which is associated with poor surgical outcomes and postoperative failure of rotator cuff repair (Shen et al., 2008; Wieser et al., 2019), but the roles of *hsa_circ_0000722* and their ceRNA interactions (*hsa_circ_0000722*-hsa-miR-129-5p-*GPD2*/*hsa_circ_0000722*-hsa-miR-30c-5p-*NCOA3*) in rotator cuff tears remain to be elucidated.

However, this study is based on a small sample of five pairs of torn supraspinatus muscle samples and matched unharmed subscapularis muscle samples, which may have limited generalizability. Therefore, key ncRNAs that found in this work need further experimental verification to confirm their potential application in rotator cuff tears.

## 4 Conclusion

We identified some ceRNA modules and important ncRNA in RCT, which may play roles in the development of RCT or rotator cuff repair. Our findings offer a new perspective on the transcriptome analysis of RCT, while pre-clinical studies followed by clinical trials are still needed to validate our findings in the future.

## 5 Materials and methods

### 5.1 Patient information

5 female patients with unilateral shoulder pain were enrolled in this study, aging 50–60. All five patients didn’t have diabetes, history of smoking, previous shoulder surgery or steroid injection. They were diagnosed with unilateral supraspinatus tears with intact subscapularis by MRI, which was then confirmed by the arthroscope. Informed consent was obtained from all subjects or their legal guardians. All methods were carried out in accordance with relevant guidelines and regulations. All experimental protocols were approved by the Ethics Committee of Peking University People’s Hospital.

### 5.2 Arthroscopic surgery and sample collection

All procedures were performed under general anesthesia. The patients were positioned in the beach-chair position and normal portals were established. The torn supraspinatus and intact subscapularis were confirmed by irrigation and debridement in intraarticular and subacromial space, then a small piece of muscle belly from each muscle was carefully collected by a grasper. After that, the supraspinatus tear was repaired using suture anchors. A total of 10 samples from five patients were stored in liquid nitrogen for further analysis.

### 5.3 Total RNA-seq library construction

Approximately 1–2 μg total RNA from each sample was used for library construction. The integrity of the total RNA was checked by agarose gel electrophoresis and the RNA concentration was quantified with a Nanodrop ND 1000 spectrophotometer (NanoDrop Technologies, United States). mRNA was enriched by NEBNext ^®^ Poly(A) mRNA Magnetic Isolation Module (NEB, United States) and ribosomal RNA (rRNA) was depleted using Ribo-Zero Magnetic Gold Kit (Human/Mouse/Rat) (Epicentre, United States). cDNA libraries were prepared using a KAPA Stranded RNA-Seq Library Prep Kit (Illumina) according to the manufacturer’s instructions. The constructed libraries were qualified by Agilent 2100 Bioanalyzer system (Agilent Technologies, CA, United States) and quantified by qPCR.

### 5.4 Total RNA sequencing

The DNA fragments in libraries were denatured with 0.1 M NaOH to generate single-stranded DNA molecules. Then the libraries were diluted to 8 nM and sequenced for 150 cycles on NovaSeq 6000 (Illumina Inc.) using NovaSeq 6000 S4 Reagent Kit (300 cycles) (Illumina Inc.).

### 5.5 RNA-seq data analysis

Quality control of RNA-seq data was carried out with FastQC (v.0.11.7) (Andrews et al., 2020). Reads were trimmed using cutadapt (v.1.17) (Martin, 2011) and aligned to human reference genome (GRCh37) using Hisat2 (v.2.1.0) (Kim et al., 2015). The differential alternative splicing events were detected by rMATS (v.4.0.1) (Shen et al., 2014). Reference-based transcriptome assembly and quantification were carried out using StringTie (v.1.3.3) (Pertea et al., 2015). The coding potential of novel transcripts was measured by CPAT software (v.1.2.4) (Wang et al., 2013). Ballgown (v.2.10.0) was applied to calculate FPKM (Fragments per kilobase of transcript per million mapped reads) values of each mRNA and lncRNA (Frazee et al., 2015). We filtered out the lowly expressed transcripts and only transcripts with average FPKM >= 0.5 in torn supraspinatus muscles or matched unharmed subscapularis muscles were selected for the subsequent analysis.

### 5.6 miRNA-seq library construction

The miRNA-seq library was constructed using NEB Multiplex Small RNA Library Prep Set for Illumina (NEB, United States). Briefly, the total RNA of each sample was used to prepare the miR sequencing library, which included the following steps: (1) 3′-adaptor ligation; (2) 5′-adaptor ligation; (3) the cDNA synthesis; (4) PCR amplification; and (5) size selection of 135–155 bp PCR-amplified fragments (corresponding to ∼15–35 nt small RNAs). Constructed miRNA-Seq library was controlled for quality using Agilent 2100 Bioanalyzer system (Agilent Technologies, CA, United States).

### 5.7 miRNA sequencing

The miRNA-seq library was denatured as single-stranded DNA molecules, captured on Illumina flow cells, amplified *in situ* as clusters and finally sequenced for 51 cycles on Illumina NextSeq 500 Sequencer according to the manufacturer’s instructions.

### 5.8 miRNA quantification

The total raw miRNA sequencing reads were filtered using a Solexa CHASTITY quality control filter. Reads were trimmed using cutadapt (v.1.14) (Martin, 2011) and aligned to the human reference genome (GRCh38) with the bowtie. Then the quantitation of miRNAs expression and novel miRNA prediction was done using miRDeep2 (v.0.0.8) (Friedlander et al., 2012). The read counts were normalized by CPM (Counts per million reads) approach. After normalization, miRNAs with average CPM >1 in torn supraspinatus muscles or matched unharmed subscapularis muscles were selected for the subsequent analysis.

### 5.9 CircRNA identification and quantification

For identification and quantification of circRNAs, reads that passed quality control were filtered to obtain trimmed data. Trimmed reads were then aligned to the human reference genome (GRCh37) using STAR (v.2.5.2b) (Dobin et al., 2013). Circexplorer2 (v.2.3.2) pipeline was used to identify the back-splice junction (circRNA) and quantify the back-splice junction reads (Zhang et al., 2016). Next, the read counts were normalized by CPM (Counts per million reads) approach. Following the analysis strategy of previous works (An et al., 2019; Wu et al., 2021; Liao et al., 2022), high confidence circRNAs were selected for subsequent differential expression analysis based on a stringent threshold of average CPM >100 in torn supraspinatus muscles or matched unharmed subscapularis muscles. The identified circRNAs were converted to circRNA ID with the web server circBase and other circRNAs were named according to the genomic locus (Glazar et al., 2014).

### 5.10 Differential expression analysis

Differential expression analysis of mRNAs and lncRNAs was performed by Ballgown (average FPKM >= 0.5 in torn supraspinatus muscles or matched unharmed subscapularis muscles, *p* < 0.05 and absolute fold change >1.5) (Frazee et al., 2014). EdgeR (v.3.20.9) (Robinson et al., 2010) was applied to identify differentially expressed miRNAs (average CPM >1 in torn supraspinatus muscles or matched unharmed subscapularis muscles, *p* < 0.05 and absolute fold change >1.5) and circRNAs (average CPM >100 in torn supraspinatus muscles or matched unharmed subscapularis muscles, *p* < 0.05 and absolute fold change >1.5). We also used GLMM model to identify differentially expressed circRNAs (Buratin et al., 2022), differential expressed circRNAs with FDR adjusted P values <0.05 were considered significant.

### 5.11 Co-expression analysis

Pearson correlation coefficient (PCC) was calculated for each lncRNA-mRNA pair and each circRNA-mRNA pair across all samples. For the co-expression networks, we only kept lncRNA/circRNA-mRNA pairs with absolute PCC >0.9. All the networks in this study were visualized using Cytoscape (v.3.7.0) (Shannon et al., 2003).

### 5.12 Functional enrichment analysis and gene set enrichment analysis

To further understand the potential functions and mechanisms of genes involved in this study, Gene Ontology (GO) enrichment analysis was performed using clusterProfiler (v.3.18.0) (Yu et al., 2012). GO terms with adjusted p-values < 0.05 were considered as significant.

### 5.13 Construction of the ceRNA network

Differential expressed mRNAs/circRNAs with uncorrected *p* < 0.05 were used to construct the ceRNA network. The regulatory relationship between miRNAs and mRNAs, lncRNAs or circRNAs were downloaded from the starbase, mirTarbase, and lncbase (Yang et al., 2011; Paraskevopoulou et al., 2016; Chou et al., 2018). We first constructed global ceRNA networks for lncRNA-mRNA pairs and circRNA-mRNA pairs, respectively, based solely on the significant sharing of miRNAs. For novel circRNAs, miRNAs targeting their host genes were considered as their regulatory miRNAs. The statistical significance of each lncRNA/circRNA-mRNA pair on sharing common miRNAs can be calculated by the hypergeometric test, which was calculated as follows:

$$p=1-\sum_{x=0}^{x-1}\frac{\begin{array}{cc} \left(\begin{array}{c} K\\ x \end{array}\right) & \left(\begin{array}{c} N-K\\ M-x \end{array}\right) \end{array}}{\left(\begin{array}{c} N\\ M \end{array}\right)}$$

Where N is the number of all human miRNAs, K represents the total number of miRNAs regulating candidate ceRNA A, M represents the total number of miRNAs regulating candidate ceRNA B, and x is the number of shared miRNAs between A and B. False discovery rate (FDR) was employed to correct the p-values, only lncRNA/circRNA-mRNA pairs with an FDR <0.05 were selected to construct the global ceRNA networks.

Then we extracted ceRNA networks consisting of only differentially expressed mRNAs, miRNAs, lncRNAs and circRNAs and calculated PCC for each lncRNA/circRNA-mRNA pair across all samples. Only lncRNA/circRNA-mRNA pairs with significantly positive correlation were retained to construct dysregulated ceRNA networks for lncRNAs and circRNAs.

### 5.14 Identification of dysregulated ceRNA modules and key non-coding RNAs

We combined the dysregulated ceRNA networks of lncRNAs and circRNAs, then utilized clusterMaker2 (v.1.3.1, https://apps.cytoscape.org/apps/clustermaker2) (Morris et al., 2011) plug-in in Cytoscape to identify network modules. In the process of identifying the module, the absolute value of the PCC was calculated for each lncRNA/circRNA-mRNA pair across all samples and it was used as the weight of edge in the network. Then ncRNAs with the highest degree and betweenness centrality were chosen as key ncRNAs (circRNAs or lncRNAs). MiRNAs that significantly regulate lncRNA/circRNA-mRNA pairs in the module were defined as key miRNAs. The hypergeometric test was used to identify these key miRNAs from differentially expressed miRNAs (p-value < 0.05, Figure 6A).

## Data Availability

The datasets presented in this study can be found in online repositories. The names of the repository/repositories and accession number(s) can be found below: https://www.ncbi.nlm.nih.gov/geo/, GSE199486.
